# Development of a Dynamically Tailored mHealth Intervention (What Do You Drink) to Reduce Excessive Drinking Among Dutch Lower-Educated Students: User-Centered Design Approach

**DOI:** 10.2196/36969

**Published:** 2022-08-11

**Authors:** Hilde van Keulen, Carmen Voogt, Marloes Kleinjan, Jeannet Kramer, Rosa Andree, Pepijn van Empelen

**Affiliations:** 1 Department of Child Health TNO (Netherlands Organization for Applied Scientific Research) Leiden Netherlands; 2 Trimbos Institute (Netherlands Institute for Mental Health and Addiction) Utrecht Netherlands; 3 Department of Interdisciplinary Social Sciences Utrecht University Utrecht Netherlands

**Keywords:** alcohol consumption, excessive drinking, lower-educated students, adolescents, young adults, dynamic tailoring, mobile health intervention, intervention mapping, health promotion

## Abstract

**Background:**

The high prevalence and adverse consequences of excessive drinking among lower-educated adolescents and young adults are public concerns in the Netherlands. Evidence-based alcohol prevention programs targeting adolescents and young adults with a low educational background are sparse.

**Objective:**

This study aimed to describe the planned process for the theory- and evidence-based development, implementation, and evaluation of a dynamically tailored mobile alcohol intervention, entitled *What Do You Drink* (WDYD), aimed at lower-educated students from secondary vocational education and training (*Middelbaar Beroepsonderwijs* in Dutch).

**Methods:**

We used intervention mapping as the framework for the systematic development of WDYD. It consists of the following six steps: assessing needs (step 1), formulating intervention objectives (step 2), translating theoretical methods into practical applications (step 3), integrating these into a coherent program (step 4), anticipating future implementation and adoption (step 5), and developing an evaluation plan (step 6).

**Results:**

Reducing excessive drinking among Dutch lower-educated students aged 16 to 24 years was defined as the desired behavioral outcome and subdivided into the following five program objectives: make the decision to reduce drinking, set realistic drinking goals, use effective strategies to achieve drinking goals, monitor own drinking behavior, and evaluate own drinking behavior and adjust goals. Risk awareness, motivation, social norms, and self-efficacy were identified as the most important and changeable individual determinants related to excessive drinking and, therefore, were incorporated into WDYD. Dynamic tailoring was selected as the basic intervention method for changing these determinants. A user-centered design strategy was used to enhance the fit of the intervention to the needs of students. The intervention was developed in 4 iterations, and the prototypes were subsequently tested with the students and refined. This resulted in a completely automated, standalone native app in which students received dynamically tailored feedback regarding their alcohol use and goal achievement via multiple sessions within 17 weeks based on diary data assessing their alcohol consumption, motivation, confidence, and mood. A randomized controlled trial with ecological momentary assessments will be used to examine the effects, use, and acceptability of the intervention.

**Conclusions:**

The use of intervention mapping led to the development of an innovative, evidence-based intervention to reduce excessive alcohol consumption among lower-educated Dutch adolescents and young adults. Developing an intervention based on theory and empirical evidence enables researchers and program planners to identify and retain effective intervention elements and to translate the intervention to new populations and settings. This is important, as *black boxes*, or poorly described interventions, have long been a criticism of the eHealth field, and effective intervention elements across mobile health alcohol interventions are still largely unknown.

**Trial Registration:**

Netherlands Trial Registry NTR6619; https://trialsearch.who.int/Trial2.aspx?TrialID=NTR6619

## Introduction

### Health and Behavioral Problem

Alcohol is one of the most commonly used substances among adolescents and young adults in the European Union. Nearly half (43.8%) of adolescents aged 15 to 19 years are current drinkers, and this prevalence increases (58%) among young adults aged 20 to 24 years. Among these groups, 51.1% (adolescents) and 54.5% (adults), drink alcohol excessively on at least one occasion per month [1]. Excessive drinking is defined as heavy drinking (ie, drinking more than the recommended weekly consumption) and binge drinking (ie, drinking more than the recommended amount on 1 occasion [2]). It is well established that excessive drinking has various negative consequences regarding health, social relationships, academic performance, and finishing school [1]. It increases the risk of (physical) violence, drunk driving, injuries, risky sexual behavior, episodic memory deficits, the development of alcohol use disorders [1,3], tissue damage in the body [4], and various forms of cancer [5].

### Excessive Drinking

In the Netherlands, various studies have shown that adolescents and young adults engage in excessive drinking [6-8]. For instance, 66% to 70% of adolescents and young adults engage in heavy drinking [8]. In the Netherlands, heavy drinking is defined as drinking more than what is proposed in the low-risk guidelines [9], in which adolescents (aged 15-17 years) are recommended to drink no alcohol and adults are recommended to drink no alcohol or, in any case, ≤1 standard drink per day. In addition, 14% to 19% of young adolescents and adults engage in binge drinking [8]. This is defined as drinking more than 4 (for women) or 6 (for men) standard alcohol units on 1 occasion at least once per week [10].

### Need for Alcohol Interventions Among Lower-Educated Students

From a public health perspective, there is an urgent need to develop and evaluate alcohol interventions that target lower-educated adolescents and young adults. When completing secondary education, students in the Netherlands can follow 3 levels of education: the lowest level is secondary vocational education and training (*Middelbaar Beroepsonderwijs* [MBO] in Dutch), the intermediate level is higher professional education (*Hoger Beroepsonderwijs* in Dutch), and the highest level is higher education (*Wetenschappelijk Onderwijs* in Dutch). In this study, we refer to students who follow a lower educational level as MBO students. MBO schools prepare students for the labor market or continuing education. In the academic year 2021-2022, there were 61 MBO schools in the Netherlands, with 504,300 students [11]. Regarding adolescents (students aged 16-18 years), 21% of the students who followed a lower educational level drank >10 glasses of alcohol on a weekend day [6]. Regarding young adults, the majority of young adults who followed a lower educational level drank alcohol in the past 12 months (63.1%), 42.7% engaged in heavy drinking, and 6.5% engaged in binge drinking [8]. Most interventions are targeted at higher-educated students [12-14]; these are mostly brief interventions, these interventions have shown modest effect sizes, and use effective techniques such as motivational interviewing, personalized feedback, identification of risk situations, and goal setting. However, interventions that effectively reduce excessive drinking among lower-educated students are still lacking, and thus, effective mechanisms that can be applied in these interventions are still largely unknown [15].

### This Study

This study aimed to describe the planned process for the theory- and evidence-based development, implementation, and evaluation of a dynamically tailored mobile intervention, entitled *What Do You Drink* (WDYD), targeting Dutch lower-educated adolescents and young adults who engage in excessive alcohol drinking. WDYD is based on the self-determination theory (SDT) [16] and self-regulation principles [17]. SDT states that autonomous motivation (ie, feeling that one’s behavior can be chosen), competence (ie, feeling confident and competent), and relatedness (ie, feeling related to and understood by others) are key targets for behavior change [18]. Regarding self-regulation, people go through the following three phases: (1) forethought, in which they prepare for behavior change via self-motivational beliefs and goal setting; (2) performance, in which they use strategies to reach goals and monitor their behavior; and (3) self-reflection, where they evaluate used strategies and learn from success or failure [17]. The developmental process is elaborated by following the six steps of the intervention mapping (IM) protocol [19], which include assessing needs (step 1), defining performance and change objectives (step 2), defining theory- and evidence-based methods and practical applications (step 3), producing the program (step 4), defining a program implementation plan (step 5), and defining a plan for process and effect evaluation (step 6). These steps are described in detail later.

## Methods

We first describe the final intervention, followed by a description of the step-by-step systematic development of the intervention.

### WDYD Intervention

WDYD is a dynamically tailored mobile intervention to reduce excessive drinking among Dutch lower-educated adolescents and young adults. WDYD is an 18-week intervention consisting of 10 sessions. It starts with a weekly session (weeks 0-5), followed by sessions every 2 weeks (weeks 7 and 9) and finally every month (weeks 13 and 17). In each session, the participants can choose from a couple of exercises that have been preselected to meet their needs, based on their activities and recordings in the app. The app consists of 39 different exercises in the form of mini-interventions and 22 brief videos in the form of role model stories. Screenshots of the intervention are provided in Figures S1-S16 in Multimedia Appendix 1.

In the first session of WDYD, the program was introduced, and users gave an estimation of their weekly alcohol consumption, received normative feedback on alcohol consumption (ie, own consumption compared with the Dutch guidelines [20]), and answered a question on whether they will take the challenge of reducing their alcohol consumption (*yes* or *no*, *I am not in the mood* or *no*, *I do not want that* or *no*, or *I am not sure I can*). Those who answer *no* receive a movie and can choose 1 of 2 mini-interventions targeting mood, motivation, or self-confidence (depending on their answer) and are asked again, after the mini-intervention, whether they want to reduce their alcohol consumption; those who answer *yes* to the first or second question are asked to plan how much they would like to drink for each day of the next week, can view a movie, and can choose 1 of 2 mini-interventions targeting planning. The session ends with a date and proposed time for the next session. Users who have set a goal to reduce their drinking are informed that they will be asked to fill out a diary daily for the next week. When users chose not to reduce their alcohol consumption, a similar intervention was repeated in the following sessions. Users who did not set a goal after 3 sessions were offered the option to continue with WDYD, take a break for 3 weeks, or stop receiving invitations for sessions.

When users chose to set an alcohol reduction goal in one of the sessions, they received daily reminders to fill out a diary to monitor their alcohol consumption (which was based on alcohol consumption of the previous day), current mood, the importance of reaching their self-set goal (to assess motivation), and perceived confidence in reaching that goal (to assess self-confidence). In addition, the follow-up sessions consisted of an overview and feedback of goal progress of the past week based on the 7 preceding diaries, the option to view a movie and to choose 1 out of 2 mini-interventions tailored to their personal situation based on their diary input (ie, when participants did not meet the goal for at least two days and mood, importance, or self-confidence had a low score), and the option to adjust their goal and consequently viewing a movie and choosing 1 of 2 mini-interventions targeting planning. In addition to the sessions, users received a notification and could select 1 of the 2 mini-interventions targeting relapse prevention (when they had not reached their goal for 3 consecutive days after having reached at least five goals). When users had not reached their goal for the last 3 consecutive days and their mood was low, they received a notification and could select 1 of 2 mini-interventions targeting mood. When users reached their goal for several days, they received positive reinforcement messages, and when they did not fill out diaries for a couple of days, they received compliance-enhancement messages. These, as well as reminders for the sessions, were offered via push notifications. In the home menu, 4 exercises were always readily available as well as 2 movies based on role modeling. For each session, participants received an invitation (at 7:30 PM or at a time chosen by participants) and 3 reminders via push notifications in case a session was not started or ended (ie, days 1, 4, and 7). The invitation for the diaries was sent via push notification at 11 AM, and a reminder was sent at 5 PM on the same day; the diary was available for 1 day, so participants were not able to fill out earlier diaries (approximately 1-minute completion time per diary).

The WDYD app consisted of 5 menus. The first menu showed participants when the next session was scheduled, the exercises they could perform, and movies they could view between sessions. The second menu showed the participants which exercises were their favorites. The third menu was the diary. The fourth menu provided an overview of alcohol intake in the week based on the diary input. Finally, the fifth menu was a profile page, in which participants could view their registration details, read a brief explanation regarding WDYD, and decide whom to contact via email in case of questions regarding the app. Screenshots of WDYD are provided in Figures S1-S16 in Multimedia Appendix 1.

### Systematic Development of WDYD

WDYD was systematically developed using IM [19], and a user-centered design [21] was applied, closely involving the target group in the intervention development. IM is used to provide a systematic and detailed description of the development of WDYD. The IM protocol provides a framework for the development of health promotion programs. It describes a step-by-step procedure that supports effective decision-making and the proper use of theory and empirical evidence. Each step has a different focus and approach for the use of evidence and theory [19].

### IM Steps

Step 1 of IM consists of a needs assessment. In this step, the health problem, behavioral and environmental causes of this problem, and related determinants are identified. In addition, the overall intervention goals, which are the desired outcomes, are defined. These health problems and causes have already been described in the *Introduction* section, and the related determinants are described in the *Results* section (IM step 2).

In step 2, the program objective and desired behavioral outcome were defined. As part of this step, the performance objectives of the behavior were formulated, functioning as the subbehaviors that underlie the program objective and behavioral outcome. Next, the changeable determinants associated with the behavioral outcome were identified. Combining the performance objectives with the determinants resulted in change objectives. Change objectives are defined as statements of what the target population should learn to change to achieve the desired behavioral outcome.

In step 3, theory- and evidence-based methods were linked to the change objectives for the behavioral outcome. Subsequently, practical applications were selected and designed to deliver theory- and evidence-based methods. Practical applications are specific techniques for the practical use of theory- and evidence-based methods in ways that fit the target group and the context in which the intervention will be delivered. The parameters for use under which the method would be effective were considered in the selection of practical applications.

In step 4, the practical applications of the intervention methods were translated into program production. The intervention materials were developed and produced in four iterations according to user-centered design to ensure it fits the needs of users [21]: (1) analyze user needs via design and concept testing, (2) test a web-based static prototype, (3) test a web-based working prototype, and (4) test the concept intervention. In each iteration, the MBO students were asked to provide input and feedback for the next iteration.

In step 5, a plan for adoption and implementation was defined, including a program that would influence the behavior of individuals who would make decisions regarding adopting and using the program. Finally, in step 6, the plan for the effect and process evaluation of the intervention is discussed [19].

### Ethics Approval

This study was approved by the Ethical Committee of the Faculty of Social Sciences of Radboud University Nijmegen (number ECSW2016-1403-390).

## Results

### Behavioral Outcome, Performance Objectives, Determinants, and Change Objectives (IM Step 1)

#### Behavioral Outcome

The objective of the program is *to reduce excessive drinking among Dutch lower-educated students aged 16 to 24 years*. The desired behavioral outcome of the WDYD intervention was formulated as follows: *Dutch lower-educated students aged 16 to 24 years drink within the normative limits of the Dutch National Health Council for low-risk drinking* [20]. This implies zero alcohol consumption for adolescents (aged 16-17 years), and for adults (aged 18-24 years), it implies a mean alcohol consumption that will not exceed 7 (for women) or 14 (for men) glasses of standard alcohol units per week and, in case of binge drinking, ≥4 (for women) or ≥6 (for men) standard alcohol units on 1 occasion after having received the intervention [10].

#### Performance Objectives

As part of the systematic development approach (IM step 2 [19]), we defined the performance objectives of the behavior (subbehaviors that underlie the program objective and behavioral outcome). The intervention targets are based on self-regulation principles [17], which break down behavioral outcomes into subgoals. Performance objectives were formulated in the order of the behavior change process, including deciding to reduce alcohol consumption, setting goals, using strategies to achieve drinking goals, and monitoring and evaluating one’s own alcohol drinking behavior. The performance objectives are presented in Table 1.

**Table 1:** Matrix of the change objectives of the dynamically tailored mobile *What Do You Drink* intervention to reduce excessive alcohol drinking among lower-educated students (aged 16-24 years) in the Netherlands.

Performance objective		Determinant			
		Risk awareness	Motivation	Self-efficacy	Social norms
1. Make the decision to reduce alcohol drinking		Acknowledge the long-term and short-term risks of alcohol drinking; be aware of responsible drinking norms	Understand misconceptions regarding positive expectancies of alcohol consumption; acknowledge the advantages of drinking less at short and long term; express the importance of drinking less or responsibly	Express confidence to reduce alcohol drinking	Experience positive norms from peers to reduce alcohol drinking
2. Set realistic drinking goals		Acknowledge the urge to set realistic drinking goals; be aware of drinking norms	Feel committed to self-set goals	Express confidence in setting realistic drinking goals	Communicate regarding realistic drinking goals with peers or buddy; ask for help from peers or buddy in setting realistic drinking goals
3. Use effective strategies to achieve drinking goals		Be aware of challenging situations	Feel motivated to achieve drinking goals	Express confidence in achieving drinking goals	Communicate regarding drinking goals with friends or peers or family or buddy; resist social pressure to drink more than the self-set goals; learn from others how they effectively reduce drinking
4. Monitor own drinking behavior		Be aware of own drinking behavior	—^a^	—	—
**5. Evaluate own drinking behavior and set goals**					
	5.1. If successful, maintain reduced alcohol drinking or adapt goal (for further reduction)	Recognize congruence and discrepancy between own drinking behavior and set goals; acknowledge the urge to maintain reduced alcohol drinking or adapt goal	Feel that reducing their goal is (socially) rewarding	Express confidence in maintaining reduced alcohol drinking or adapt goal	—
	5.2. If unsuccessful (lapse), revise used strategies in reducing alcohol drinking or adapt drinking goal	Recognize congruence and discrepancy between own drinking behavior and set goals	Feel motivated to revise used strategies to reduce alcohol drinking; feel motivated to adapt drinking goal after lapse	Express confidence to deal with tempting or challenging situations; express confidence to manage negative feelings	Learn from others how they cope with lapses and revise used strategies to reduce alcohol drinking and adapt drinking goals

^a^Not available (missing data; there were no change objectives made for the combination of this performance objective and determinant).

#### Determinants

A systematic literature search resulted in 4 changeable determinants of excessive drinking among adolescents and young adults: risk awareness, motivation, self-efficacy, and social norms. These are related to the key targets of SDT for behavior change (ie, autonomous motivation, competence, and relatedness) [16,18]. First, those who drink excessively usually underestimate their amount of drinking as well as the short- and long-term effects of excessive drinking [22-24]. According to the precaution adoption process model [25], awareness of the risk involved in performing a particular behavior is crucial in taking the first step toward behavioral change. Therefore, increasing risk awareness may be an effective mechanism for reducing alcohol consumption.

Second, intrinsic motivation is a risk factor for excessive drinking among young adults [26]. A Dutch study targeting alcohol reduction among adolescents and young adults was not effective for lower-educated students because they were particularly unmotivated to change their behavior [27]. This was probably due to the focus on higher-educated students and those ready to reduce excessive drinking in the intervention. Increased motivation to drink alcohol also influences drinking behavior. For instance, a study showed that motivations to drink alcohol that included positive outcomes of drinking (eg, socializing and having fun) was related to more drinking [28]. Increasing intrinsic motivation to reduce drinking through an intervention that suits the needs of the target group and decreasing motivation to drink alcohol by focusing on socially evoked alcohol drinking might be effective mechanisms for behavioral change. On the basis of the SDT [16], intrinsic motivation is a key factor in the adaptive self-regulation of behavioral change. In addition, increasing intrinsic motivation to reduce drinking among adolescents and young adults who drink excessively is shown to decrease alcohol consumption [29,30]. Therefore, increasing intrinsic motivation may be an effective mechanism for reducing alcohol consumption.

Third, self-efficacy is related to alcohol drinking [26]. Young adults with low self-efficacy to refuse alcohol drinking are more likely to engage in heavy drinking [31]. Drinking refusal self-efficacy is shown to be affected by mood [32]. A negative mood seems to contribute to a lowered drinking refusal self-efficacy, resulting in more maladaptive drinking behaviors, such as uncontrolled excessive drinking [33]. High perceived self-efficacy in refusing alcohol drinking is shown to result in less high-risk drinking behaviors [34]. In addition, drinking refusal self-efficacy is affected by self-efficacy expectations regarding dealing with social pressure. Thus, enhancing self-efficacy may be an effective mechanism for reducing alcohol consumption.

Feeling confident in dealing with social pressure leads to a decrease in alcohol consumption and binge drinking [27]. Social norms, the fourth determinant, also comes into play. Social pressure plays a role in excessive drinking among young adults [35,36], with social norms perceived by peers in particular [24,37]. Thus, not only feeling more confident in coping with social pressure but also addressing the social norms that correspond with this pressure could function as changeable factors in decreasing excessive alcohol consumption. Thus, enhancing self-efficacy may be an effective mechanism for reducing alcohol consumption.

#### Change Objectives

To define the change objectives, the performance objectives were combined with the determinants, resulting in specific goals that would enhance the behavior change process during the intervention (eg, *express confidence to reduce alcohol drinking*). Table 1 depicts the matrix of the performance objectives, with the change objectives specified per determinant in the WDYD intervention. Regarding risk awareness (determinant), when making the decision to drink less alcohol (performance objective 1), an example of a change objective is that one should be aware of the short- and long-term risks of alcohol drinking and of responsible drinking norms. Regarding intrinsic motivation (determinant), to set realistic drinking goals (performance objective 2), an example of a change objective is that one should feel committed to their self-set goals. Regarding self-efficacy (determinant), when using effective strategies to achieve drinking goals (performance objective 3), an example of a change objective is that one should express confidence in achieving drinking goals. Regarding social norms, when using effective strategies to achieve drinking goals (performance objective 3), an example of a change objective is that one should communicate their goals with others or learn from others how they effectively reduce drinking.

### Selection of Methods and Practical Applications (IM Step 3)

#### Theory- and Evidence-Based Methods

Next, we selected theory- and evidence-based methods and their corresponding practical applications for the intervention (IM step 3) [19]. Multimedia Appendix 2 provides an overview of the theory- and evidence-based methods that we selected based on the change objectives presented in Table 1. Dynamic tailoring, or just-in-time adaptive intervention (JITAI), was selected as the overarching method. It ensures not only personalization of feedback to the individual but also adaptation based on variations in time. In other words, methods can be tailored to the situation of a person based on psychological and behavioral changes over time [38,39]. Dynamic tailoring is based on the principle of timing of the right type and amount of support. This requires monitoring of the individual. Dynamic tailoring or JITAI has been more effective than nondynamic tailoring or non-JITAI [39,40]. The parameters for use under which tailoring is effective are that the intervention is tailored to determinants related to behavior change or relevance (eg, age, sex, and alcohol consumption). These were applied; the determinants are described in IM step 2, and the messages are tailored to users’ age, sex, and current alcohol consumption based on input from the diaries.

We used theory- and evidence-based methods derived from self-regulation [17] theories as the broad umbrella, as these target performance objectives directly, including self-monitoring [41], behavioral feedback, and goal setting [42]. These methods have been found to be effective in reducing alcohol consumption [12,43]. Furthermore, these self-regulatory methods can cause participants to see the discrepancy between their alcohol drinking goal and behavior, leading to increased risk awareness. Other methods applied to increase awareness include normative feedback and personalized risk information [44].

To increase motivation, we used methods based on motivational interviewing and self-reward. Motivational interviewing [45] is an effective method for alcohol reduction interventions targeting adolescents and young adults [14,46]. Self-reward has not yet been properly examined as an effective method for changing behavior [47], but it has shown promising effects on promoting behavior change and maintenance of physical activity [48] and fruit consumption [49].

To improve self-efficacy in coping with difficult alcohol-related situations, we included methods such as coping planning and relapse prevention. Problem solving is associated with a reduction in excessive alcohol consumption [50], which includes coping planning and relapse prevention [51]. Self-efficacy was also increased through self-persuasion [52] and stress management [53]. Self-persuasion can reduce alcohol consumption compared with direct persuasion (ie, providing arguments) [54]. Research has shown that stress management can reduce negative emotions and alcohol consumption among students [55].

To address social norms, modeling and social support were incorporated into the intervention. Modeling [56,57] has been shown to be effective when normative information on the alcohol consumption of peers (eg, negative attitude toward excessive drinking) is given [58]. Social support [59] has not yet been properly examined as an effective method for reducing alcohol consumption. In the context of alcoholics anonymous, it has been suggested as an effective mechanism in promoting a sober lifestyle [60] and has also been shown to be an effective method in various health behavior change interventions [61].

#### Practical Applications

The practical application of the chosen methods as part of WDYD is presented in Multimedia Appendix 2. Here, we provide examples of the practical applications and the parameters for use under which the methods are effective. First, we provide 2 examples regarding performance objective 1 (*making the decision to reduce drinking*). To increase users’ awareness of possible drinking norms (change objective), targeting the determinant *risk awareness*, the method *normative feedback* will be applied keeping the parameter for use in mind, that is, the feedback needs to be individual, follow the behavior in time, and be specific [62]. In the practical application, users will receive information regarding their own alcohol consumption compared with alcohol drinking guidelines [20] (Figure S1 in Multimedia Appendix 1). To target the determinants *motivation and self-efficacy*, the method *motivational interviewing* was used by means of several practical applications in the app while maintaining the parameters for use, such as a supportive relationship with the client combined with the evocation of change talk in a manner that is collaborative, evocative, autonomy supporting, and exploring rather than confronting, educating, authoritative, and explanative [62]. The practical applications of this are a value clarification, importance and confidence rulers, identifying personal strengths, and looking forward and back. For example, to allow users to express the importance of drinking less (change objective for determinant *motivation*), the practical application *values clarification* was used to explore among users important values and to evoke change talk by examining among users how these fit with reducing their alcohol consumption. In addition, upon the interest of the user, examples of values among peers and how these values relate to reduced drinking were provided (Figures S2-S5 in Multimedia Appendix 1).

Second, 2 examples of practical applications for performance objective 2 (*set realistic drinking goals*) are described. App users chose their alcohol drinking goals based on their preferences and abilities to achieve their goals. Users were encouraged to set smart goals that were specific, measurable, achievable, relevant, and time bound. The app provided different exercises to achieve these drinking goals. To enhance expressing confidence in setting realistic drinking goals (change objective), targeting the determinant *self-efficacy*, the method *goal setting* was practically applied by asking users to set drinking goals based on their consumption during the previous week and checking whether the set goals are realistic (Figure S6 in Multimedia Appendix 1). The reference period (ie, alcohol consumption in the past week) and checking whether the set goals were realistic were included to adhere to the parameters for use of goal setting (ie, users feel committed to their goal, and goals are difficult but available within the individual skill level [62]. To stimulate communication regarding realistic drinking goals with peers or a buddy and to ask for help in setting realistic drinking goals by peers or a buddy (change objectives), targeting the determinant *social norms*, the method *mobilize social support* was chosen. The parameter for the use of mobilizing social support is, that support for behavior change is positive and includes caring, trust, openness, and acceptance, [62]. This was practically applied by stimulating users to choose a buddy among their friends who could help them reduce alcohol consumption and to specify how they can help (Figures S7-S10 in Multimedia Appendix 1).

A total of 3 examples of practical applications with regard to performance objective 3 (*use effective strategies to achieve drinking goals*) are described. To promote resistance to social pressure to drink more than the self-set goals and learning from others how they effectively reduce drinking (change objectives), targeting *self-efficacy*, the method *modeling* was practically applied by showing videos and role model stories with tips and tricks from peers on how they reduce alcohol drinking and how they say no to alcohol, keeping in mind the parameters for use of modeling (ie, the user can identify with the model, and the model needs to be a coping model who provides their own solutions [62] (Figures S11 and S12 in Multimedia Appendix 1). To promote feeling motivated to achieve drinking goals, targeting the determinant *motivation*, the method *self-reward* was practically applied by an exercise focusing on how and when to reward yourself. The parameter for use is that self-praise or self-reward is prompted if there has been effort or progress in performing the behavior [63]. This was applied by asking users to select a behavioral achievement (eg, reaching their goal for alcohol consumption for 1 day, 3 days, or a week) and to link a reward to this achievement.

Regarding performance objective 4 (*monitor own drinking behavior*), an example of a practical application of the method *self-monitoring* to enhance awareness of one’s own drinking behavior (change objective), targeting the determinants *risk awareness* and *motivation*, was an alcohol drinking diary, along with a mood diary. The parameters for the use of self-monitoring are that the monitoring must be of a specific behavior, that the data must be interpreted and used, and that the reward must be reinforced to users [62]. This was applied by prompting users to fill in their diary on a daily basis, specifying how many glasses of alcohol they drank yesterday and how they are feeling today. They could also indicate how important they think it is to reach their alcohol goal for that day and how much confidence they have in reaching that goal. Users received a notification for the diary at 11 AM and a reminder at 5 PM when they did not fill in their diary to promote the use of the dairy. The app contained a separate page where users were able to see their diary and progress regarding their alcohol drinking goals. If users reached their drinking goals for a couple of days in a row, a positive reinforcement of progress toward goals was prompted (Figures S13 and S14 in Multimedia Appendix 1).

With regard to performance objective 5 (*evaluate own drinking behavior and set goals*), 2 examples of practical applications are provided. To promote recognition of congruence and discrepancy between own drinking behavior and set goals (change objective), targeting the determinant *risk awareness*, the method *behavioral feedback* was applied. The parameter for use is that feedback needs to be individual, follow the behavior in time, and be specific [62]. This was applied by showing the user the discrepancy between the set goals and drinking behavior in glasses per day of the past week in a graph (Figure S15 in Multimedia Appendix 1). To stimulate users expressing confidence in maintaining reduced alcohol consumption or adapting goals (change objective), targeting the determinant *self-efficacy*, the methods *relapse prevention* and *planning coping responses* were applied. The parameter for use is that high-risk situations are identified and coping responses are practiced. This was applied with exercises focusing on learning from lapses (eg, drinking too much on 1 occasion) and how to get back on track. For example, in an exercise, users are stimulated to identify high-risk situations (ie, where, with whom, their thoughts, feelings, and consequences) and to learn coping responses (ie, helping thoughts and responses). They can also see an example of a peer who describes a high-risk situation and plans a coping response.

### Program Production of WDYD (IM Step 4)

WDYD was systematically and iteratively developed in 4 phases with the active involvement of lower-educated students to examine what fits their needs (IM step 4) [19], following user-centered design principles [21]. WDYD was developed by the Research Institute for Applied Sciences (Nederlandse organisatie voor Toegepast-Natuurwetenschappelijk Onderzoek), Trimbos Institute, and Radboud University Nijmegen, and the software for WDYD was developed by Newstory. Students from 2 MBO schools participated in the iterative development of WDYD. They were recruited via a contact person at each school (ie, a teacher). The students involved in the development were diverse in terms of sex, age, and alcohol consumption. Their inputs were included in the following version and were consecutively pretested. Participants received a gift voucher as a reward for their participation in the pretests. An advisory board of relevant stakeholders (Trimbos Institute, Dutch MBO Council, MBO’s Nova College and Deltion College, Dutch Foundation Testjeleefstijl, Open University Heerlen, Maastricht University, and Radboud University Nijmegen) offered recommendations for the program design, implementation, and evaluation. WDYD was developed in 4 phases and pretests. The aim of the pretests was to evaluate the preferences of the students, that is, their needs and requirements for the program and their preferred strategies with regard to layout and future use (eg, channel, frequency, and duration). After each pretest, feedback was processed in the prototype, and the prototype was further developed based on recommendations.

The first pretest of WDYD was a concept test consisting of a scenario of the program and mock-ups of possible strategies used for improving motivation, self-efficacy, mood, and planning and for preventing relapse. This concept was tested through 4 focus group interviews with students who engaged in excessive drinking (N=23 students). The mean age of the participants was 17 (range 15-21) years, 52% (12/23) were male, and they varied according to study type (social care: 6/23, 26%; car engineering: 5/23, 22%; audiovisual: 3/23, 13%; aviation and service: 3/23, 13%; legal service: 2/23, 9%; photography: 2/23, 9%; executive secretary: 1/23, 4%; and enforcement: 1/23, 4%). The concept was positively evaluated with a mean grade of 7.1 on a 10-point scale (1=very bad to 10=excellent; range 6.5-8.5). Students were positive regarding the variety of methods used and the tone of voice in that they experience an autonomous choice to reduce their drinking. They perceived the Dutch guidelines [9] (no alcohol for adolescents and maximum 1 standard drink per day for adults) as somewhat unrealistic, and most students indicated that they did not want to reduce alcohol consumption. They experienced feelings of shame in using a program to reduce their alcohol consumption and did not feel the need to ask for help to reduce their consumption; they indicated that it is important that WDYD can be used individually and privately, and they suggested that an app may be a good solution for that. Participants indicated that privacy in the use of the program is crucial, as it accounts for the experience of freedom or autonomy of choice (with regard to the decision to reduce alcohol drinking as well as which program parts they want to use and when). Suggestions for further development were to enhance personal relevance (eg, information regarding the purpose and target group of the program, key focus on coping with challenging situations and empowerment instead of therapeutic focus, and information regarding the consequences of alcohol drinking), language (ie, avoiding therapeutic language and explaining difficult terms, such as binge drinking), guarantee privacy of use (ie, preference for the program to be used via an app and not wanting to share program content with others), and duration and frequency (ie, they wanted to spend a maximum of 15 minutes per week on the program; this may be a couple of times per week to, for example, monitor drinking; they wanted to use it until they drank less or reached their goal). This concept test resulted in a production document in which the features, functionalities, and requirements of WDYD were stated based on the students’ recommendations on the first prototype.

The second pretest consisted of the evaluation of a web-based, static prototype in 5 focus group interviews, in which the prototype was evaluated individually (students could scroll through the pages via a link on their phone and write down their notes), both individually and classically (the prototype was presented via a beamer), or vice versa. These mock-ups were presented as several pages, illustrating the process of navigating through the app. The questions focused on the practical applications of the behavior change methods in the app (eg, exercises, diaries, and notifications) and the design. The sample consisted of 57% (33/58) male students, the participants’ mean age was 18.4 (range 16-22) years, and they drank on average 10 glasses of alcohol per week (range 0-56 glasses). More than half of the sample (30/58, 52%) engaged in binge drinking at least once a week and drank >7 glasses of alcohol weekly (30/58, 52%). The app was evaluated with a mean grade of 7.0 on a 10-point scale (1=very bad to 10=excellent; range 4-8.5). Students were positive regarding the design, user-friendliness, and monitoring of their drinking behavior. The app was evaluated as very user intuitive: it was clear what they needed to do and how they could do it. Some of the exercises were unclear, especially the titles, pictures, or icons, and the texts were evaluated as too long. Although we explained difficult words (ie, binge drinking), students struggled to read them, so they were better avoided. In addition, participants preferred use of pictures, icons, and shortened texts. They also suggested using videos for more variety and providing them with notifications for completing the diary. They recommended reducing the amount of text, explaining some exercises more clearly, using more and better suitable pictures, and using swiping to navigate through the different pages of the exercises (instead of the next or previous page buttons). These recommendations were processed into a third prototype of the app.

The third pretest consisted of an evaluation of a web-based working prototype of the app via an individual interview (N=3 students). Participants were asked to download the app on their phone and use it daily for a couple of days (including the weekend). After this, they were called for an evaluation. The questions were focused on unclarities, navigation, content, usability, and overall impression. A total of 67% (2/3) of the students were male, the mean age was 17.3 (range 16-18) years, they drank an average of 9 glasses of alcohol per week (range 5-12 glasses), 67% (2/3) of the students drank >7 glasses per week, and no participants engaged in binge drinking (ie, 2 of the male students drank 5 glasses per day on 2 consecutive days of the weekend). The prototype was positively evaluated with a mean grade of 7.3 on a 10-point scale (1=very bad to 10=excellent; range 6-8). The students were positive regarding the design, navigation, language, and interactive nature of the app. They were also positive regarding the ease of use and time for participation. They suggested further reducing the amount of (informative) texts and improving the visibility of the role model videos. These recommendations were processed in the fourth prototype of WDYD.

The fourth pretest consisted of an evaluation of the full product (ie, the working WDYD app) in individual interviews (N=4 students). Students used the prototype in the form of a mobile app for at least eight days so that they could have received the baseline questionnaire, 2 sessions, and notifications to fill out their diary daily. After this period, they were called for an evaluation. The interviews focused on the usability and content of the app. The mean age of the sample was 18.3 (range 17-19) years, and 50% (2/4) of the students were male. They drank on average 5.3 glasses of alcohol weekly (range 3-8), 25% (1/4) drank >7 glasses per week, and 25% (1/4) engaged in binge drinking at least once a week. The app was evaluated very positively with a mean grade of 8.2 (1=very bad to 10=excellent; range 7.8-8.5). At the start, students had somewhat low expectations of the app, but they were positively surprised: they were positive regarding informativity, design, user-friendliness (ie, ease of use and time needed to participate), language (ie, understandable), notifications (amount and type), and freedom of choice they experience within the app (eg, the option to view a video and the option to choose 1 out of 2 exercises). The number of notifications was evaluated as good, but there was room for improvement: 1 participant received the notification although they had already filled in the diary, and 2 participants received a reminder although they had already filled in the diary; this needs to be improved in the next version. The preference for the use of the weekly overview (ie, a graphical overview of their alcohol consumption of the week based on the diary input) varied; some participants evaluated it as informative, whereas others preferred the use of the diary as informative in itself and indicated that a weekly overview was not needed. The weekly overview was maintained in the diary, and users could decide for themselves whether to use this overview. The feedback was perceived as somewhat brief, but this was not changed because they only experienced 2 out of the 10 sessions, and during the preceding pretests, the amount of feedback was perceived as too much.

Suggestions for improvements were processed into the final version of WDYD, which was used for the randomized controlled trial.

### Implementation Plan of WDYD (IM Step 5)

To ensure successful implementation (IM step 5) [19] of WDYD, the target group was intensively and iteratively involved in the developmental process of the intervention, following user-based design principles [21]. Students from 2 MBO schools participated in 4 different pretests (refer to the Program Production of WDYD subsection). In addition, 2 classes of MBO students in media design developed short videos in which role models (ie, students) provided tips and tricks to reduce their alcohol consumption and to deal with challenges. These videos were developed in sprint sessions (writing a plan, developing scripts, and refining the videos), in which students received feedback from their teacher and 2 developers of WDYD and made refinements to the plan, scripts, and videos. This resulted in 22 videos that were incorporated into WDYD as part of several exercises.

The intervention was developed and implemented in collaboration with the Trimbos Institute. The Trimbos Institute was the owner of WDYD from the start, as they manage the knowledge network for e- and m-mental health in the Netherlands. This enhanced the opportunity for a broad implementation of WDYD and integration in interventions already used by the Trimbos Institute nationally.

Several strategies have been implemented to specifically stimulate the sustained use of WDYD. Notifications were used to invite and remind participants to fill in their diary, participate in a session, and keep up with their alcohol drinking goals (positive reinforcement messages). This way of prompting a review of progress toward goals is associated with a reduction in alcohol consumption frequency [58]. In light of behavioral maintenance, motivational interviewing was applied to establish a working alliance and to increase intervention engagement, intrinsic motivation, and confidence for change [45]. WDYD not only consisted of several motivational interviewing exercises (such as values clarification, importance and confidence ruler, identifying personal strengths, looking forward, and looking back) but also applied principles of motivational interviewing. These principles were autonomy support (ie, by providing participants as much choice as possible, eg, to provide them with the chance to plan the time to the next session, to provide the choice to view a video, and to choose 1 out of 2 exercises), partnership (ie, the input of participants in the diaries and sessions was the point for departure of the sessions, and participants were stimulated to provide their opinion or solution in the exercises), acceptance and compassion (ie, feedback via push notifications or sessions were written empathetically and personal, without coercion or blame), and evocation (ie, the point of departure for feedback within WDYD was the input of the participant in the diary and sessions; the exercises included in WDYD were interactive, and participants were encouraged to elaborate on their own motivation, confidence, planning strategies, mood, and relapse prevention strategies). In addition, dynamic tailoring was implemented to elicit behavior change, but it was also used to prevent intervention fatigue and, therefore, induce sustained use [15,38] by withholding from intervening when a user is not receptive to the intervention (eg, when a user does not fill in the diary repeatedly, no exercise will be offered).

### Evaluation Plan of WDYD (IM Step 6)

To evaluate the effectiveness of WDYD (IM step 6) [19], a study protocol was written, and a 2-arm randomized controlled trial was planned (trial registration: Netherland Trial Registry NTR6619 [64]). The goal was to randomly assign participants to the intervention group (ie, WDYD intervention) or the control group (ie, no intervention). The participants were recruited via a web-based lifestyle monitor for MBO students [65]. Students who drank alcohol excessively according to the monitor were invited to participate in the study. The eligibility criteria for participation in the study were (1) drinking excessively, that is, drinking more than the low-risk drinking guidelines recommended for adolescents (aged 16-17 years) to drink no alcohol and for adults to drink a maximum of 1 (women) or 2 (men) glasses of standard alcohol units daily, or (2) binge drinking, that is, drinking ≥4 (for women) or ≥6 (for men) standard alcohol units on 1 occasion [20]. In addition, participants had to be aged ≥16 years at the time they signed up, have to be computer or internet literate, and have to provide their informed consent before participation in the study.

The primary outcome measures were excessive drinking (yes or no), binge drinking (yes or no), and the mean weekly alcohol consumption. The secondary outcome measures were intrinsic motivation and self-confidence toward reducing alcohol consumption and the mood of the participant. Outcomes were assessed at baseline, week 9, and week 33 by using web-based surveys and ecological momentary assessments [66]. The ecologic momentary assessments will consist of brief daily diary measurements, assessed every 6 weeks for 7 consecutive days, ending after 33 weeks (ie, weeks 1, 7, 13, 19, 25, 31, and 33 after baseline). Participants in the intervention group will evaluate WDYD in a web-based survey at week 9 after baseline, and they will receive questions on overall acceptability (mean grade), usability of the app, information supplied, and design principles behind the app.

## Discussion

### Principal Findings

This study provides a comprehensive and detailed description of the rationale and plan for the development, implementation, and evaluation of WDYD, a dynamically tailored mobile health intervention. WDYD was developed to reduce excessive drinking among lower-educated adolescents and young adults in the Netherlands. The IM protocol was followed for the development of WDYD [19]. Furthermore, a user-centered approach was applied in designing the intervention [21].

### Contribution of IM

Poorly described interventions, or *black boxes*, have long been criticized in the eHealth field [67,68]. Treatment rationales within empirical papers are often briefly described in the methods section, where the theoretical framework, delivery, behavior change techniques, and content of the intervention are addressed to a limited extent [19]. The IM approach resulted in a systematically developed intervention by using theory-driven and evidence-based methods. The proper description of behavior change techniques and their practical applications in WDYD contributed to the replicability of the intervention. Furthermore, it can inspire researchers to design new interventions [69].

WDYD was developed in response to an earlier web-based, brief, single-session version of WDYD. This older version showed nonsignificant results for lower-educated adolescents and young adults (aged 16-24 years) and pointed out specific recommendations for improvement [27]. For instance, the new version of WDYD included exercises for students not ready to reduce excessive drinking and implement known conditions for the effectiveness of motivational interviewing (eg, autonomy support, evoking change talk, and partnership) and a nonjudgmental tone of voice [45]. The new version of WDYD was a longer and more intensive intervention, which included multiple sessions to facilitate improvement in intrinsic motivation for change as well as goal striving and persistence [17]. It was based on effective interventions using these strategies [70,71]. The IM protocol is useful for revising and updating existing interventions and provides an opportunity to account for earlier study flaws and limitations.

### User-Centered Design

A user-centered design is crucial to maximize the fit of the intervention with the target group [72]. The user-centered design approach resulted in the intensive involvement of the target group in the developmental process of WDYD. The 4 pretests demonstrated how involving the target group led to an increasingly suited intervention, resulting in more positive evaluations of the app. After every pretest, the app was adjusted according to the feedback and recommendations of the target group. This led to changes in the design that would have been easily missed if the target group was not involved in the developmental process. For instance, the pretests indicated that students struggled with difficult wordings (eg, binge drinking), so they were avoided as much as possible. Another example was that students preferred the use of brief textual feedback in the app, alternate textual feedback with pictures and icons, and enhanced variability by including videos. Therefore, we decided to let students develop videos themselves, which included the role model stories of students who provided tips to reduce their alcohol intake and deal with challenges. In addition, textual feedback was rewritten in a terse style, and pictures and icons were added.

### Limitations

There are some limitations regarding the developmental process of WDYD. First, the sample sizes of the 2 final pretests were small. Moreover, not all pretests included excessive drinkers only, as the focus of these tests was on evaluating usability and intuitive use. This may explain why participants in the final 2 pretests evaluated the app as more positive (mean grades of pretests 3 and 4 were 7.3 and 8.2, respectively, compared with pretests 1 and 2, which were 7.1 and 7.0, respectively). Therefore, the results of the final 2 pretests should be interpreted cautiously. However, having only a handful of students in the qualitative evaluation allowed for in-depth interviews, addressing all facets of the app. Second, the final pretest indicated some flaws with regard to the notifications (eg, notifications for the diary were sent although participants had already filled in the diary). This was resolved and thoroughly tested before implementation in the trial. Third, lower-educated students appeared to be a very challenging target group; on the one hand, students indicated in the pretests that they preferred a brief intervention, to spent a maximum of 15 minutes of their time for the intervention per week, that the textual feedback was as brief as possible, and that the intervention included as much freedom of choice as possible. Therefore, we provided students with several options (eg, viewing a video and choosing 1 out of 2 tailored exercises). On the other hand, as an intervention developer, intervention time is essential for inducing behavior change, and providing users with freedom of choice increases the risk of lower exposure. In addition, personal relevance of the app remained a problem throughout the pretests; even in the fourth pretest, the expectations of students of the app were low, but when participating, they were surprised by the app and perceived it as informative and supportive.

### Conclusions

It can be concluded that IM and user-centered design are useful means to develop an evidence-based and theory-driven intervention to reduce excessive drinking among lower-educated students. The target group of lower-educated students who drink alcohol excessively needs more attention in future research to design well-suited and effective interventions.

## Appendix

Multimedia Appendix 1 Screenshots of the *What Do You Drink* intervention.

Multimedia Appendix 2 Methods and practical applications of WDYD.

## Abbreviations

IM: intervention mapping
JITAI: just-in-time adaptive intervention
MBO: Middelbaar Beroepsonderwijs
SDT: self-determination theory
WDYD: *What Do You Drink*
