# Supplementary material for: Development of a Dynamically Tailored mHealth Intervention (What Do You Drink) to Reduce Excessive Drinking Among Dutch Lower-Educated Students: User-Centered Design Approach
Source: JMIR Form Res. 2022 Aug 11;6(8):e36969. doi: 10.2196/36969 (PMC9412899; doi:10.2196/36969)

### Multimedia Appendix 1. Screenshots of the WDYD intervention

(Figure S1) Normative tailored feedback (FigureS2) Exercise ‘values clarification’ (1)


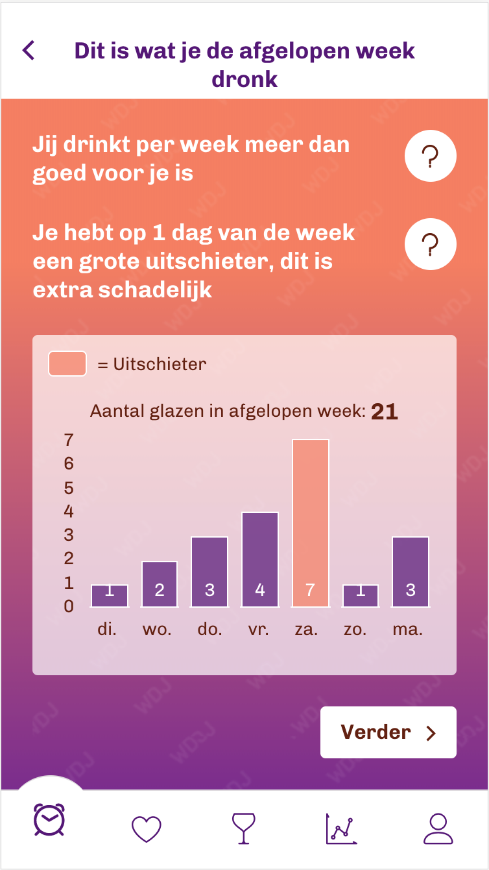

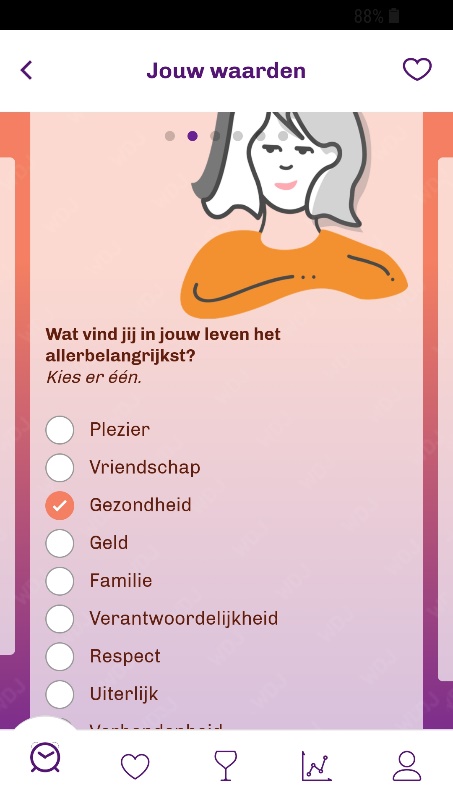


(Figure S3) Exercise ‘values clarification’ (2) (Figure S4) Exercise ‘values clarification’ (3)


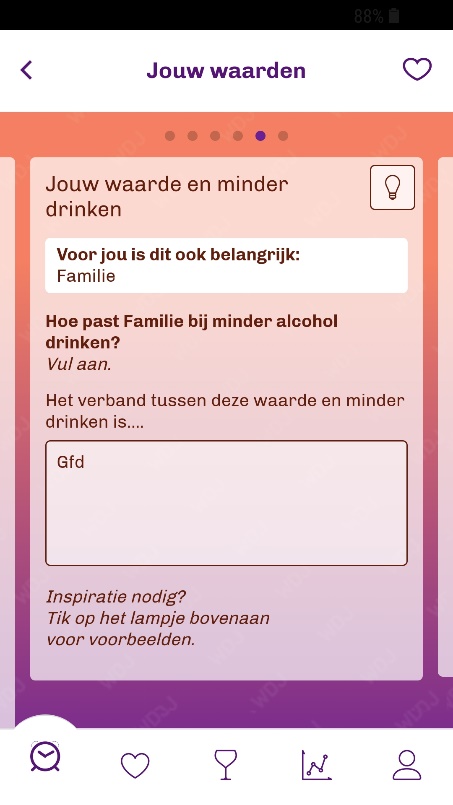

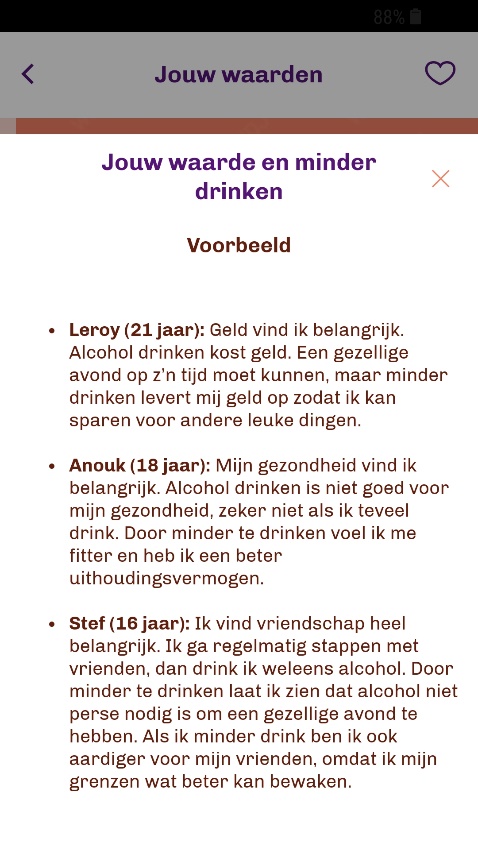


(Figure S5) Exercise ‘values clarification’ (4) (Figure S6) Goal setting


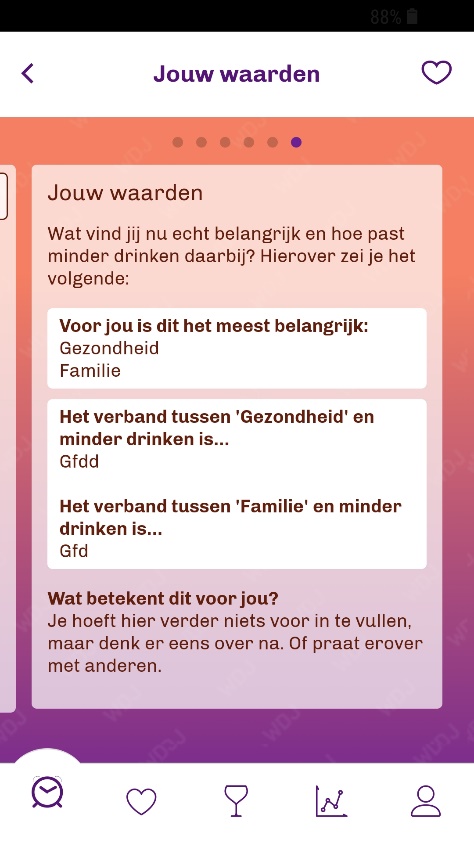

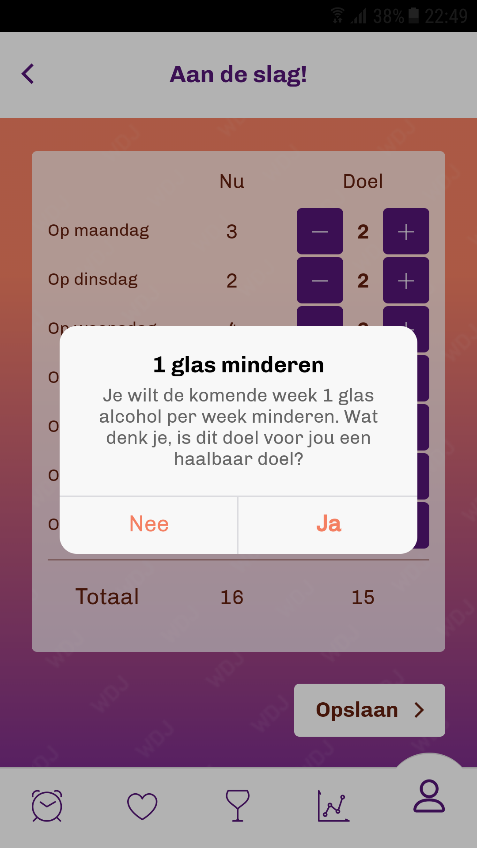


(Figure S7) Exercise ‘ask a buddy support’ (1) (Figure S8) Exercise ‘ask a buddy support’ (2)


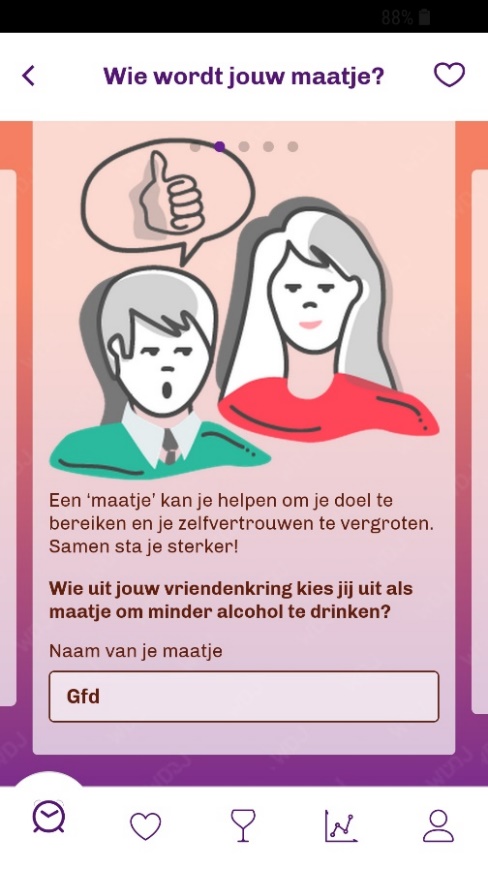

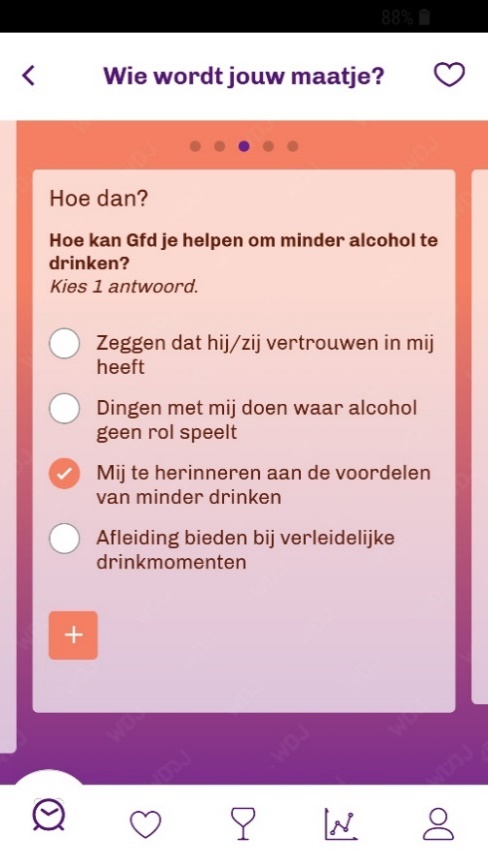


(Figure S9) Exercise ‘ask a buddy support’ (3) (Figure S10) Exercise ‘ask a buddy support’ (4)


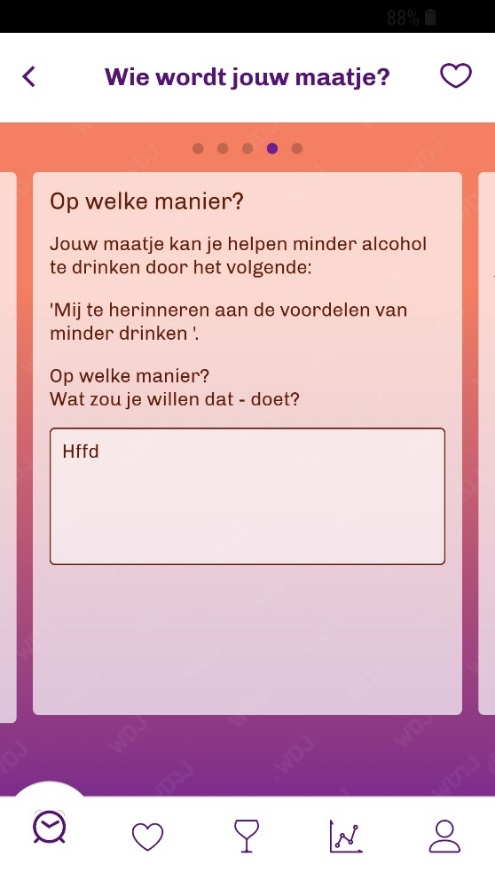

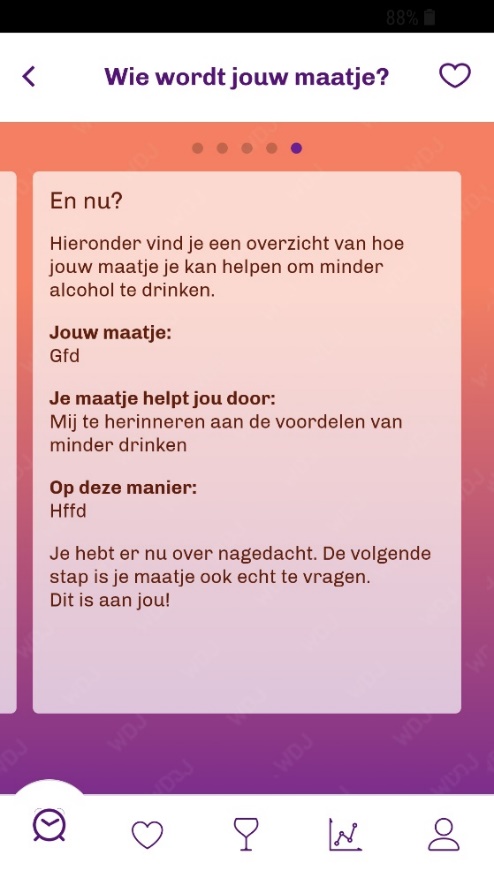


(Figure S11) Role model video (Figure S12) Role model story


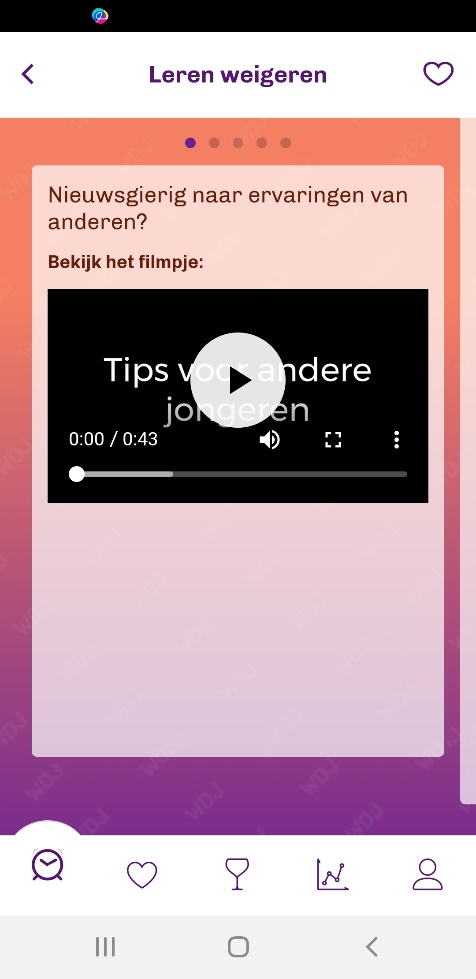

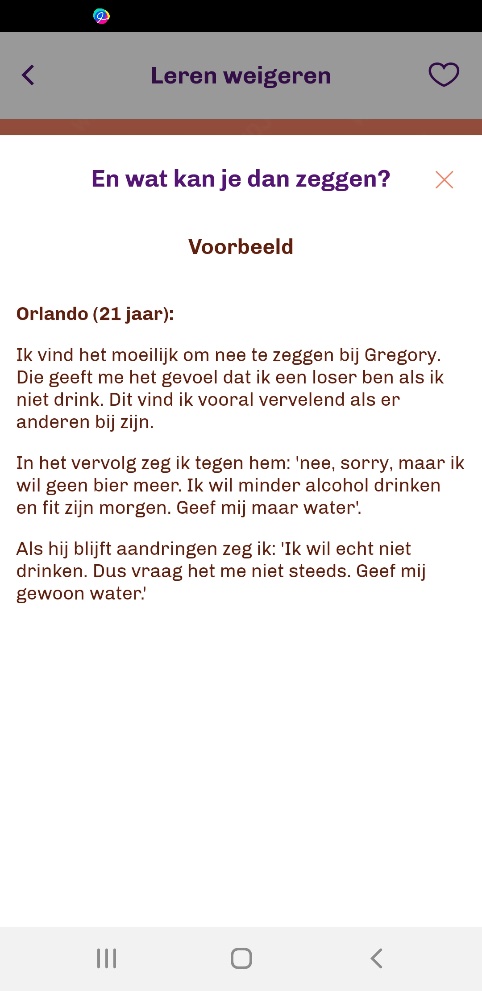


(Figure S13) Notification and diary (3^rd^ menu) (Figure S14) Positive reinforcement with regard to goal achievement


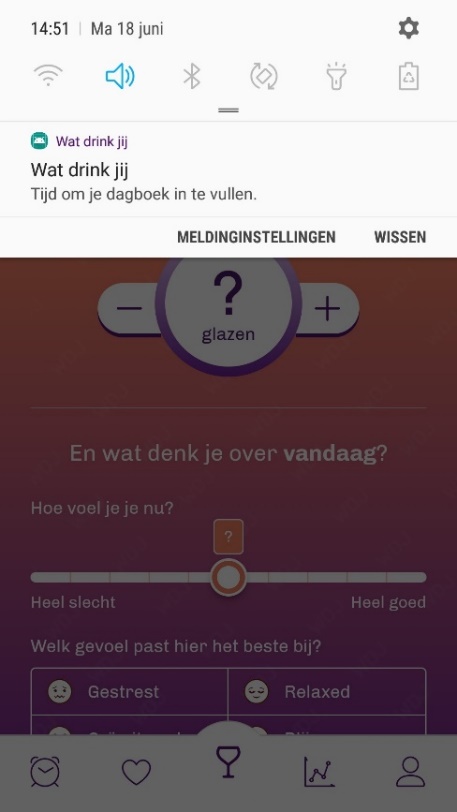

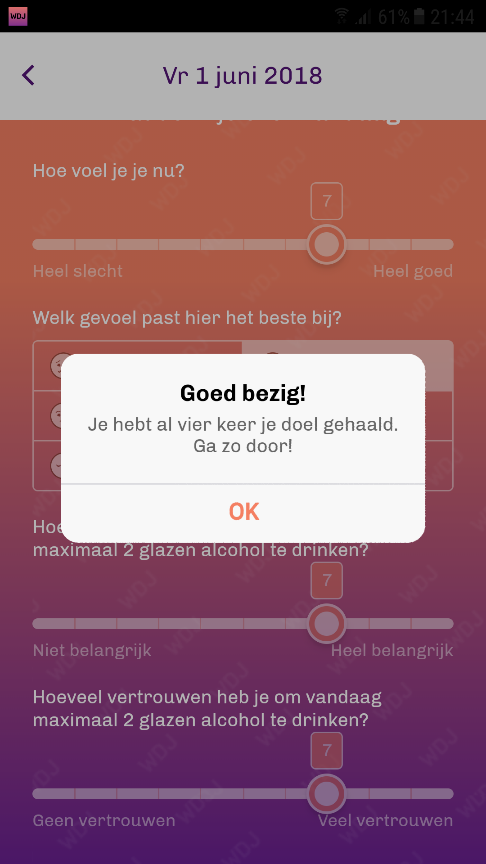


(Figure S15) Fourth menu (overview of (Figure S16) Motivational exercises offered

alcohol intake)


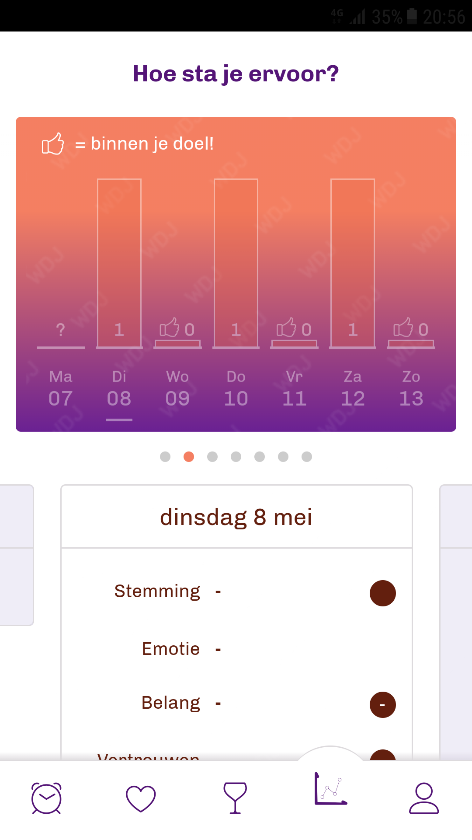

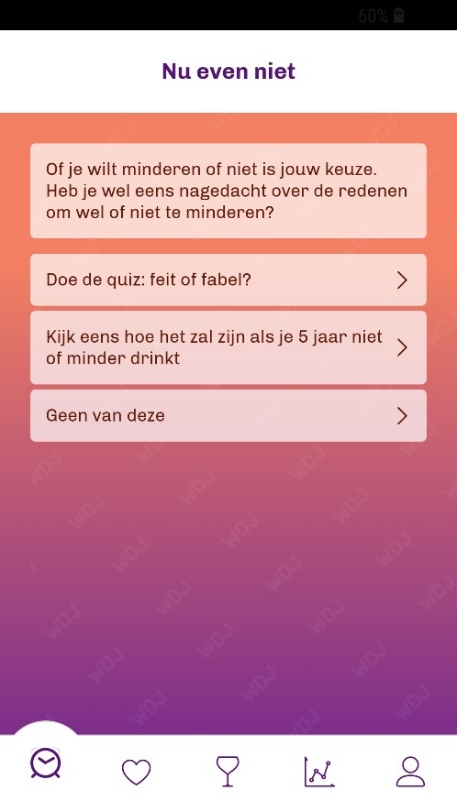


(Figure S17) WDYD session: (Figure S18) First menu (planning for next session,
tailored feedback and small exercises and movies readily available in between sessions).


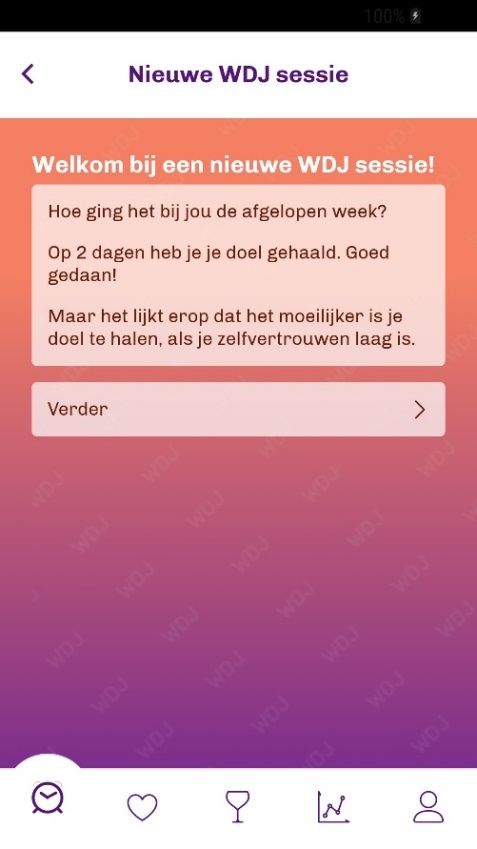

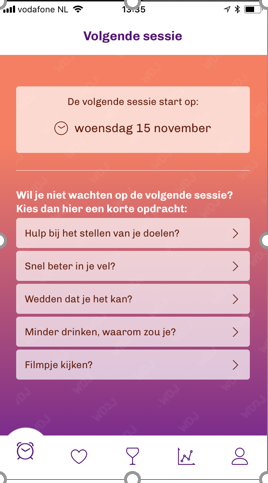

Supplement: Multimedia Appendix 1 [file formative_v6i8e36969_app1.docx]
