# Supplementary material for: Development of a Dynamically Tailored mHealth Intervention (What Do You Drink) to Reduce Excessive Drinking Among Dutch Lower-Educated Students: User-Centered Design Approach
Source: JMIR Form Res. 2022 Aug 11;6(8):e36969. doi: 10.2196/36969 (PMC9412899; doi:10.2196/36969)
Supplement: Multimedia Appendix 2 [file formative_v6i8e36969_app2.docx]

### Multimedia Appendix 2. Theory- and evidence-based methods and practical applications for the dynamically tailored mobile What Do You Drink intervention to reduce excessive drinking among lower-educated students (aged 16-24 years) in the Netherlands (Table S1).

| Performance objective and determinants | | Theory- and evidence-based methods and their definition | Practical applications |
| --- | --- | --- | --- |
| 1. **Make the decision to reduce alcohol drinking** | | | |
|  | Risk awareness | - Normative feedback (ie, monitor and provide informative or evaluative feedback on performance of the behavior in comparison with guidelines or peers) - Personalized risk information (ie, provide information regarding personal costs or risks of action or inaction with respect to target behavior) [44] | - After users filled in their weekly alcohol consumption data, normative feedback will be provided regarding their alcohol consumption compared with the alcohol drinking guidelines [20]. - Personalized risk information will be provided based on responses to a screening test that assesses users’ name, sex, age, and alcohol use. It provides information regarding the adverse health effects that are linked to their drinking behavior. |
|  | Motivation | - Motivational interviewing (ie, a collaborative, goal-oriented style of communication with attention to language of change and strengthen motivation for and commitment to a goal by evoking the person’s reasons for change in an atmosphere of acceptance and compassion) [45] | - Motivation is increased through means of value clarification and an importance ruler. Users choose what they find important in life and relate this to their alcohol consumption. |
|  | Self-efficacy | - Modeling (ie, providing an appropriate model being reinforced for the desired action) [56,57] - Self-persuasion (ie, users persuade themselves to change their attitudes or behavior) [52] | - Role models state why it is important to not drink excessively or to reduce drinking in a video or in role model stories. - An exercise will let users advise themselves in what the first step is in reducing their alcohol consumption and how to take this step. - Self-efficacy is increased via several motivational interviewing exercises, such as the confidence ruler, and identification of personal strengths and how to use this in less drinking. |
|  | Social norms | - Motivational interviewing (ie, a collaborative, goal-oriented style of communication with attention to language of change and strengthen motivation for and commitment to a goal by evoking the person’s reasons for change in an atmosphere of acceptance and compassion) [45] - Modeling (ie, providing an appropriate model being reinforced for the desired action) [56,57] | - Role models tell regarding their negative attitude regarding excessive drinking and positive attitude toward reduced drinking though videos and role model stories. |
| 1. **Set realistic drinking goals** | | | |
|  | Self-efficacy | - Goal setting (set or agree about a goal defined in terms of the behavior to be achieved) [42,62] | - Drinking goals are set based on alcohol consumption of the last week. When goals are confirmed, a notification will pop up, checking whether the set goals are realistic. Goal-setting exercises will help the user in setting realistic goals (eg, the exercise *make goals small*). |
|  | Social norms | - Mobilize social support (ie, prompting communication regarding behavior change to provide instrumental or emotional social support) [59] | - Users will be encouraged to choose a buddy to reduce alcohol drinking together. |
| 1. **Use effective strategies to achieve drinking goals** | | | |
|  | Risk awareness | - Personalized risk information (ie, provide information regarding personal costs or risks of action or inaction with respect to target behavior) [44] | - An exercise will explain the impact of tempting situations on reaching alcohol reduction goals and helps users to gain insight into which situation leads to drinking for them specifically. |
|  | Motivation | - Self-reward (prompt self-praise or self-reward if there has been effort or progress in behavioral performance) [63] | - Self-reward is prompted in exercises teaching how and when to reward yourself |
|  | Self-efficacy | - Planning coping responses (ie, prompting users to list potential barriers and ways to overcome these) [51] - Stress management (ie, practicing cognitive and behavioral techniques with the goal to manage and cope with stress) [53] | - A volitional help sheet including implementation intentions [68] will guide users in coping with tempting alcohol situations and cravings - Exercises with cognitive behavioral theory components (eg, helpful thinking) will help users to enhance their mood and reduce negative emotions. For example, helpful thinking was applied to support users in coping with negative feelings that lead to alcohol drinking. Another example is an exercise to plan nice things with others to promote positive emotions. |
|  | Social norms | - Modeling (ie, providing an appropriate model being reinforced for the desired action) [56,57] - Mobilize social support (ie, prompting communication regarding behavior change to provide instrumental or emotional social support) [59] | - Role models provide tips regarding how to say no to alcohol drinking and explain how they prevent drinking excessively. - Users are instructed to tell their peers regarding their alcohol reduction goals so that peers can support them in reaching their goals. |
| 1. **Monitor own drinking behavior** | | | |
|  | Risk awareness/motivation | - Self-monitoring (ie, prompting the person to keep a record of a specified behavior) [41] | - The app will contain a diary, where users can fill in how much alcohol they drank the other day and how they are feeling that day. |
| 1. **Evaluate own drinking behavior and set goals** | | | |
|  | Risk awareness | - Behavioral feedback (ie, monitor and provide informative or evaluative feedback on performance of the behavior) [62] | - Users review the discrepancy between set goals and drinking behavior. |
|  | Self-efficacy | - Relapse prevention and planning coping responses (ie, prompting users to list potential barriers and ways to overcome them) [51] | - To prevent relapse, the app provides exercises where users are taught to learn from lapses (eg, drinking more than the set goal). |
|  | Social norms | - Modeling (ie, providing an appropriate model being reinforced for the desired action) [56,57] | - To prevent relapse, a role model shows what they have learned from a lapse and how to cope with this. |
|  | Motivation | - Motivational interviewing (ie, a collaborative, goal-oriented style of communication with attention to language of change and strengthen motivation for and commitment to a goal by evoking the person’s reasons for change in an atmosphere of acceptance and compassion) [45] | - Therapeutic alliance [50,58,69], identify and use personal strengths, or learn from past successes. |
